# Supplementary figures and images for: Combined genome-wide association study of 136 quantitative ear morphology traits in multiple populations reveal 8 novel loci
Source: PLoS Genet. 2023 Jul 17;19(7):e1010786. doi: 10.1371/journal.pgen.1010786 (PMC10351707; doi:10.1371/journal.pgen.1010786)

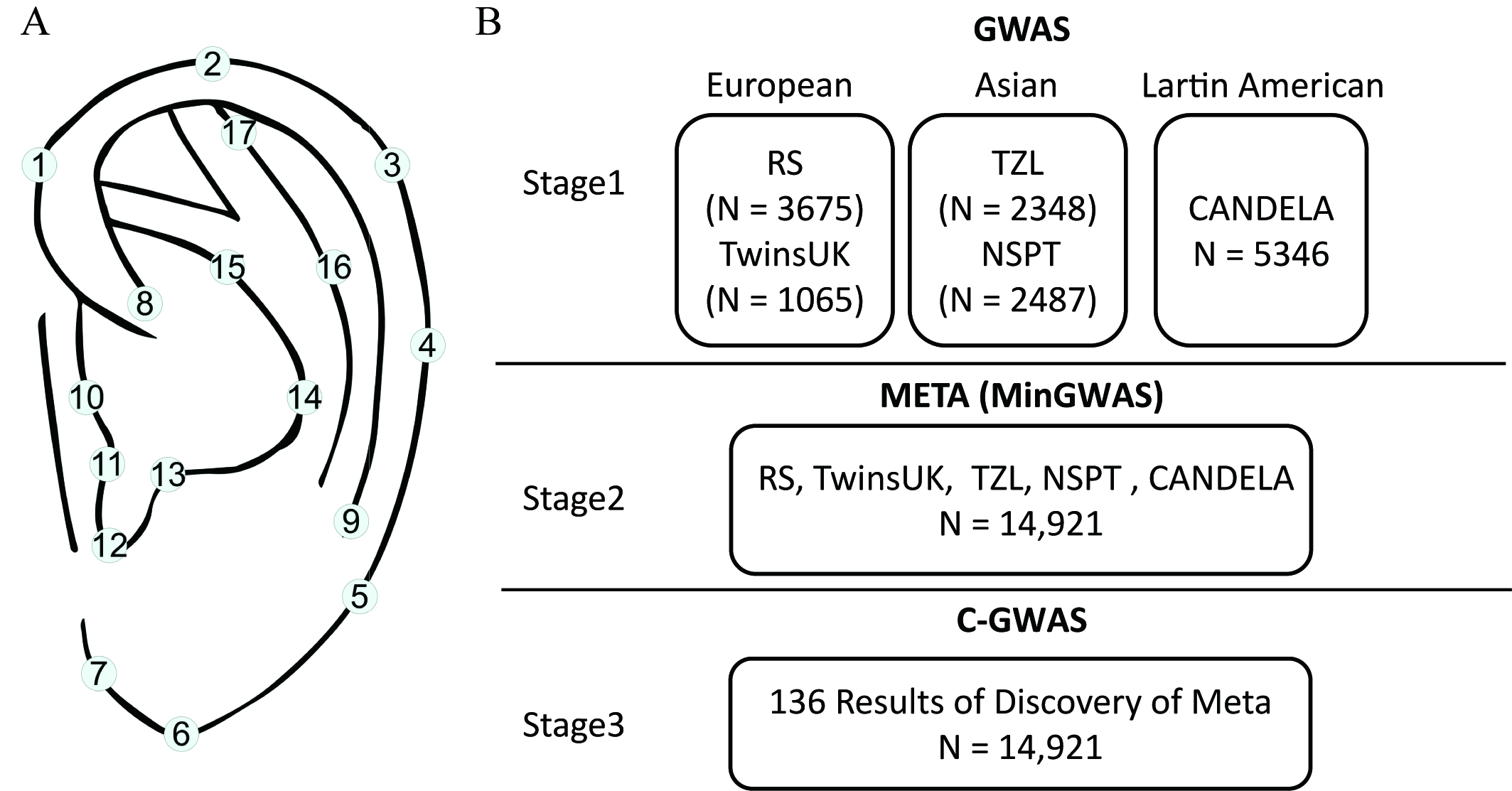

Supplement: S1 Fig — (A) The location of selected 17 ear landmarks. (B) Design of the current study. (TIF) [file pgen.1010786.s001.tif]

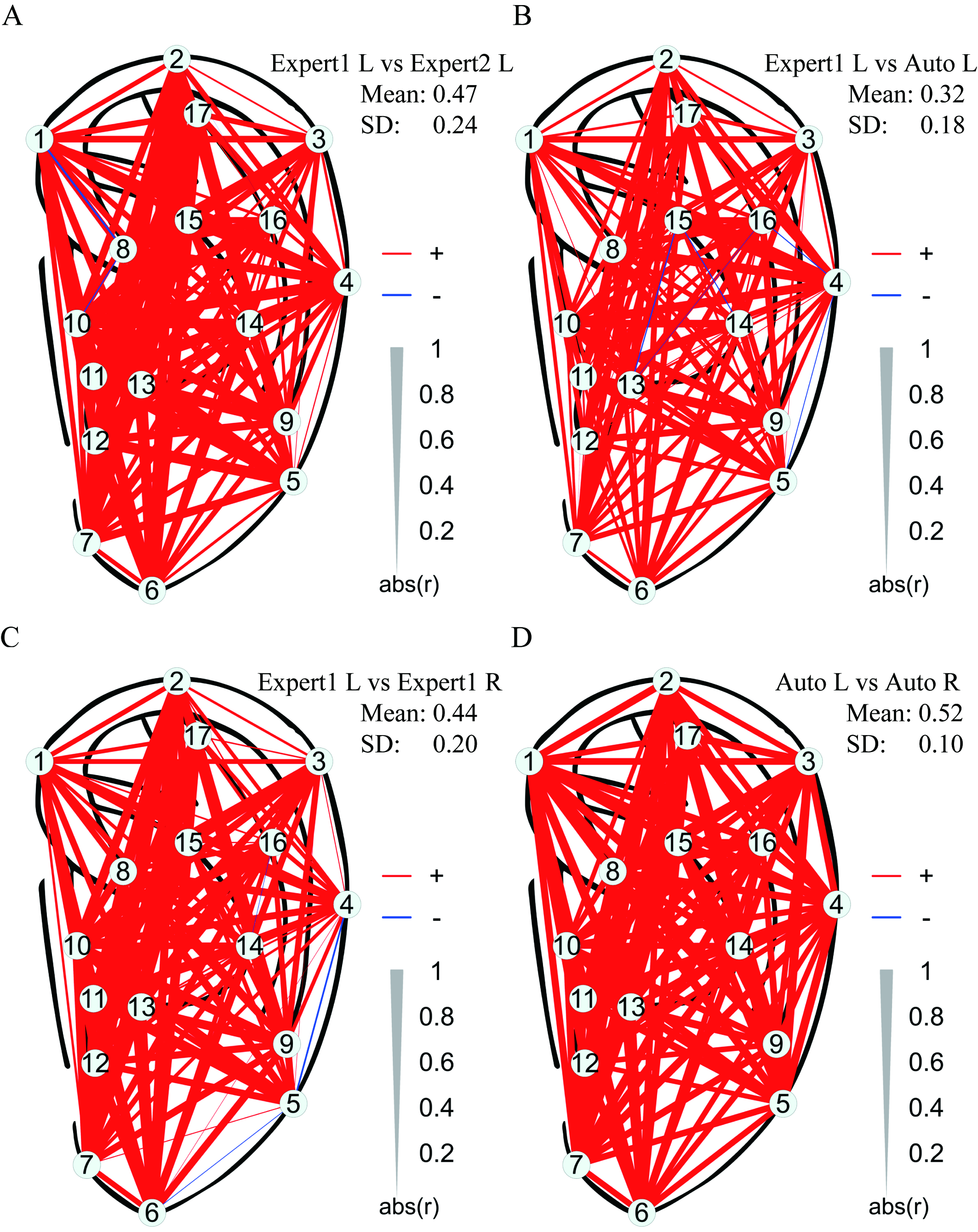

Supplement: S2 Fig — (A) correlation between left ear phenotypes by manual-landmarking (expert 1 vs. expert 2). (B) correlation between right ear phenotypes by manual-landmarking and auto-landmarking (expert 1 vs. auto). (C) correlation between phenotypes from left and right ears by manual-landmarking. (D) correlation between phenotypes from left and right ears by auto-landmarking. (TIF) [file pgen.1010786.s002.tif]

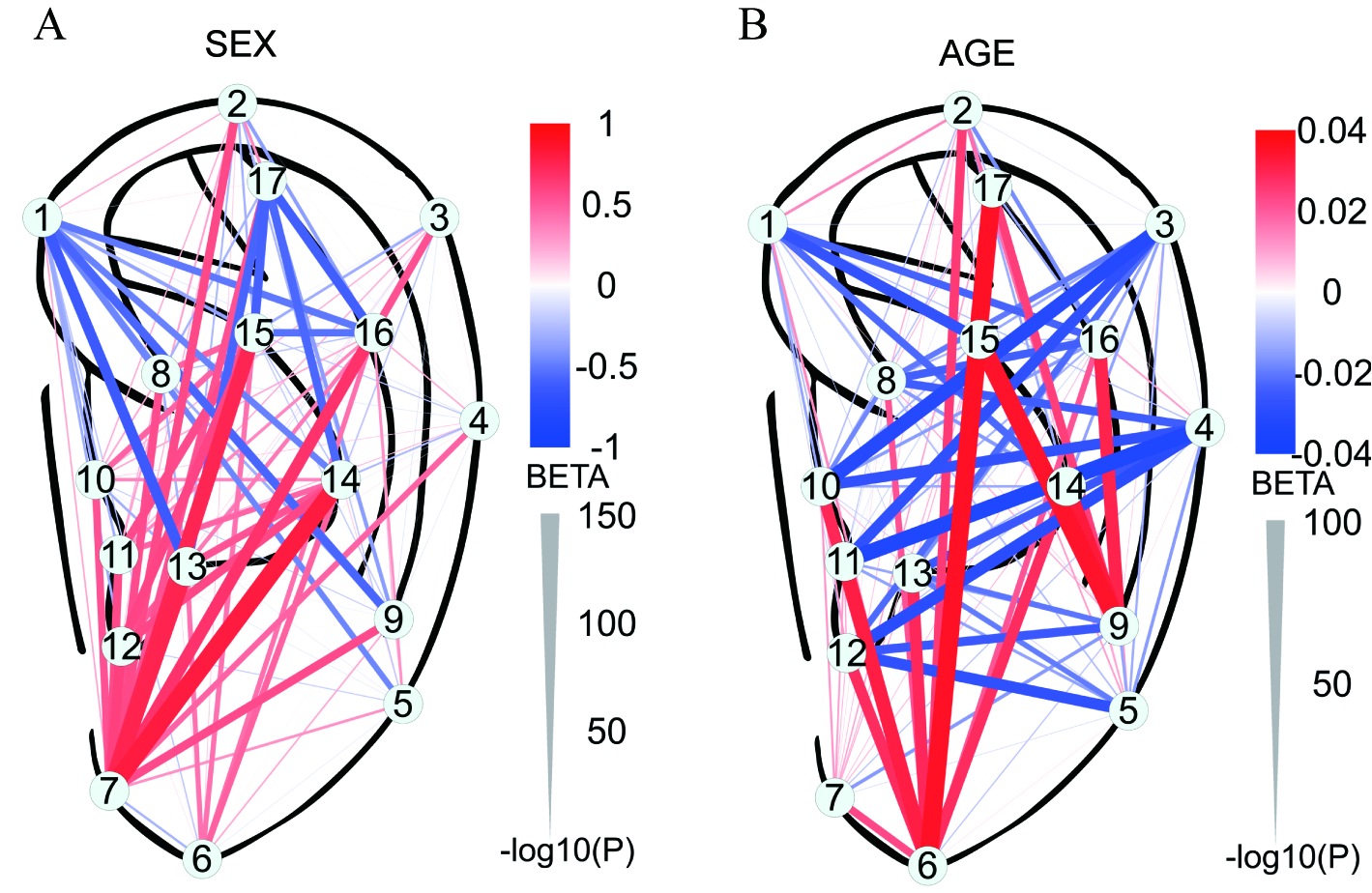

Supplement: S3 Fig — Please note the different figure legends in these two figures. (TIF) [file pgen.1010786.s003.tif]

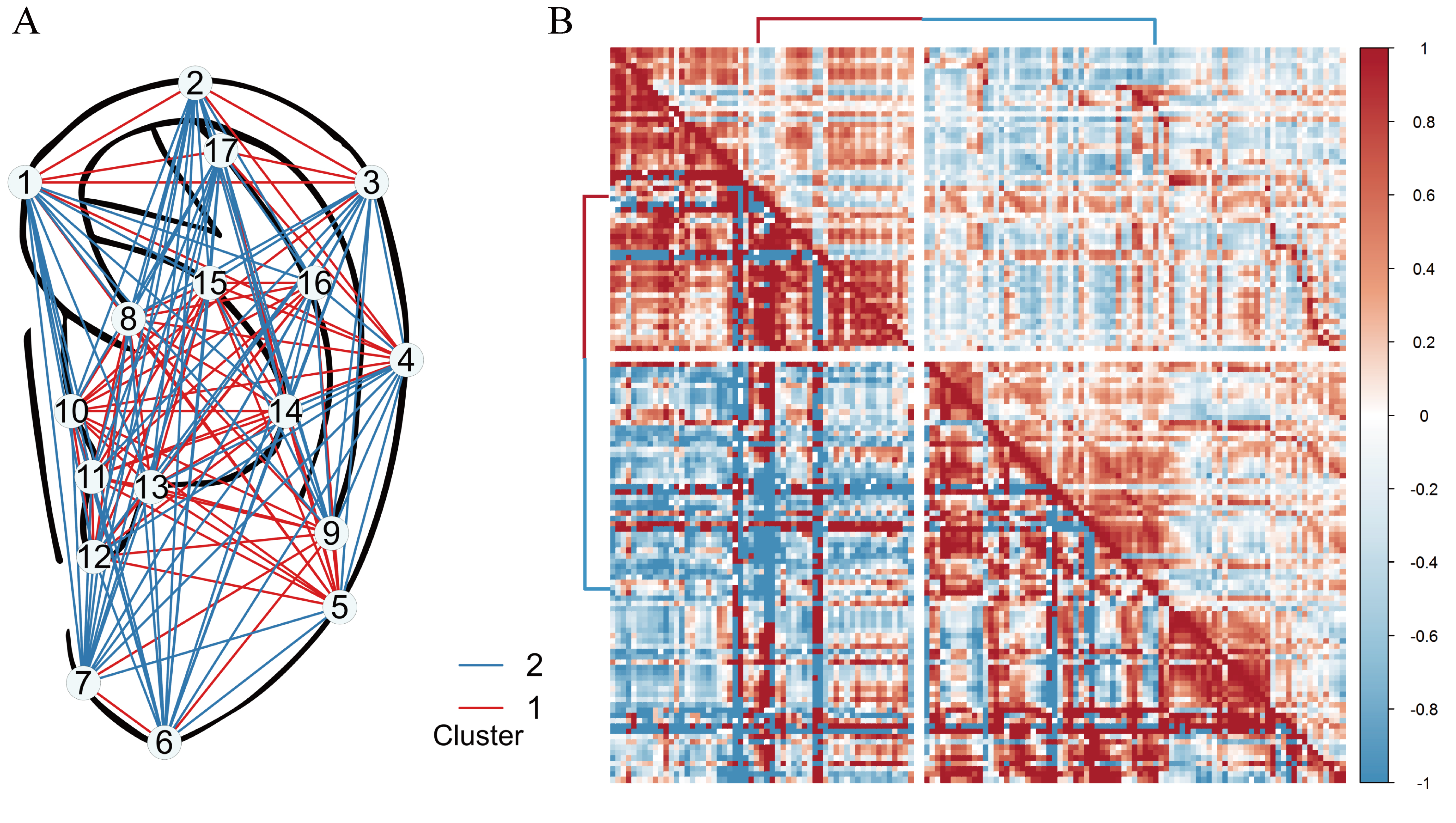

Supplement: S4 Fig — (A) Two clusters for 136 phenotypes. (B) Phenotypic (right up) and genetic correlation matrix (left down) within and between the cluster. (TIF) [file pgen.1010786.s004.tif]

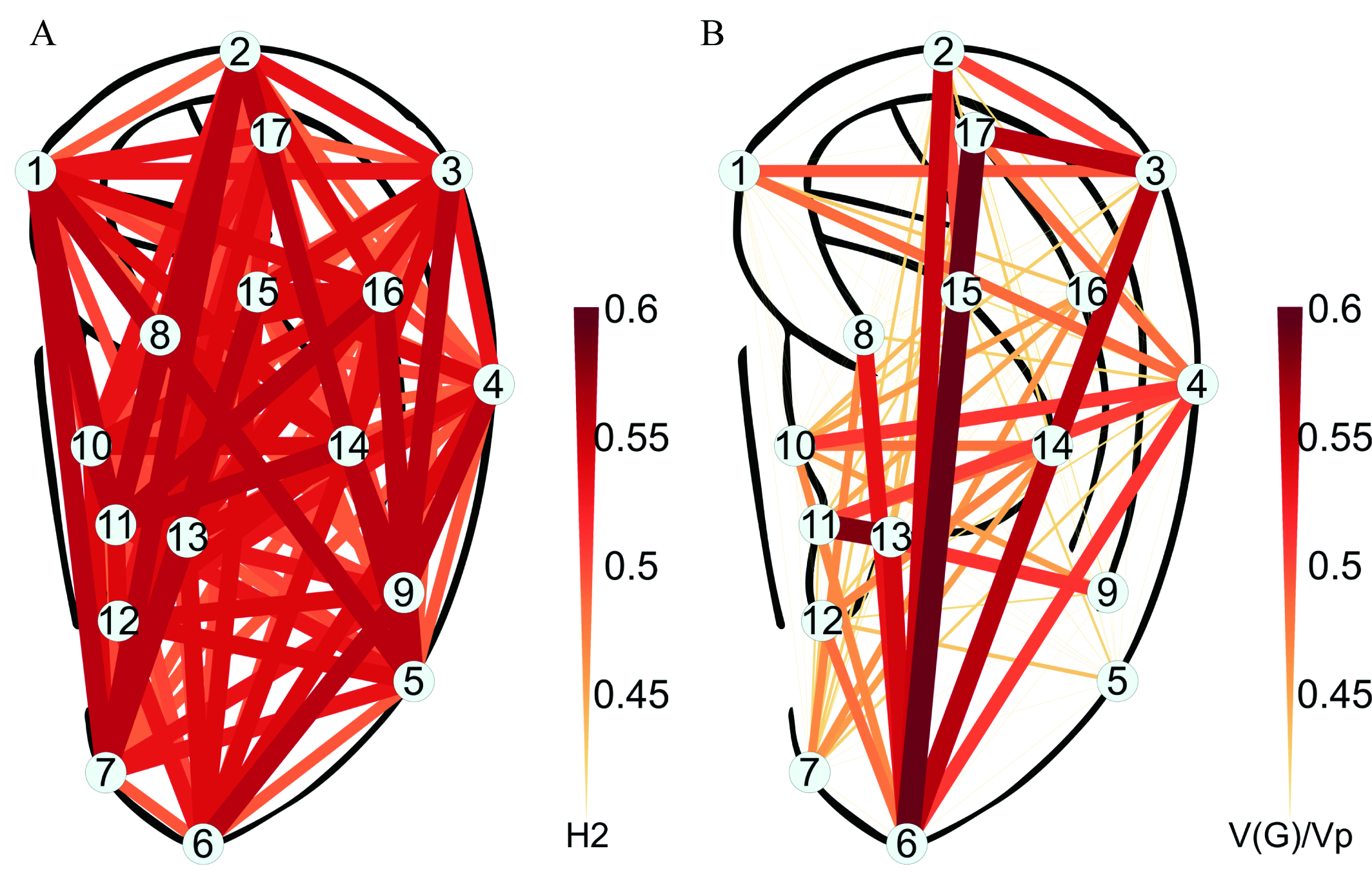

Supplement: S5 Fig — (A) Twin heritability. (B) SNP-based heritability. (TIF) [file pgen.1010786.s005.tif]

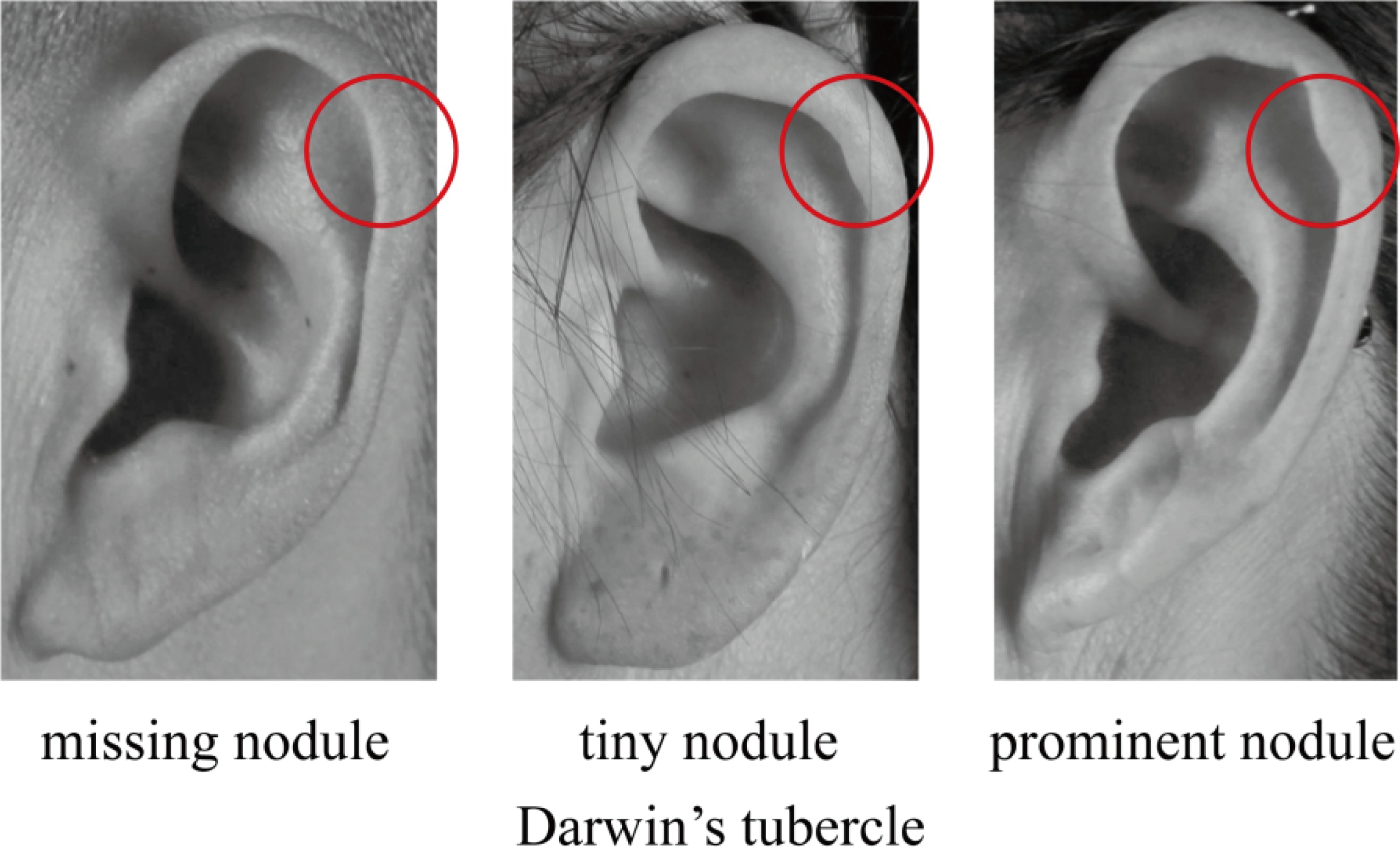

Supplement: S7 Fig — (TIF) [file pgen.1010786.s007.tif]

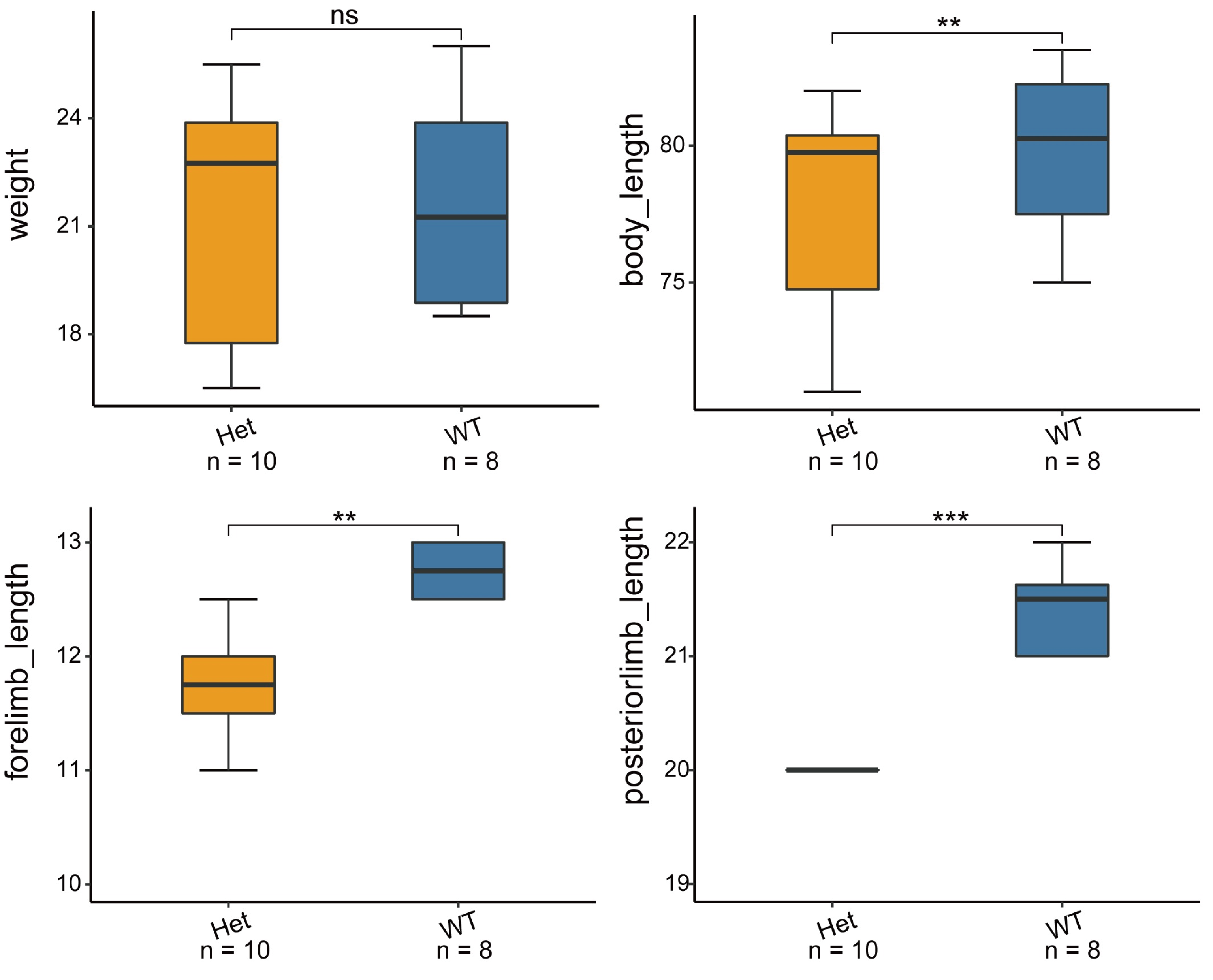

Supplement: S8 Fig — (TIF) [file pgen.1010786.s008.tif]

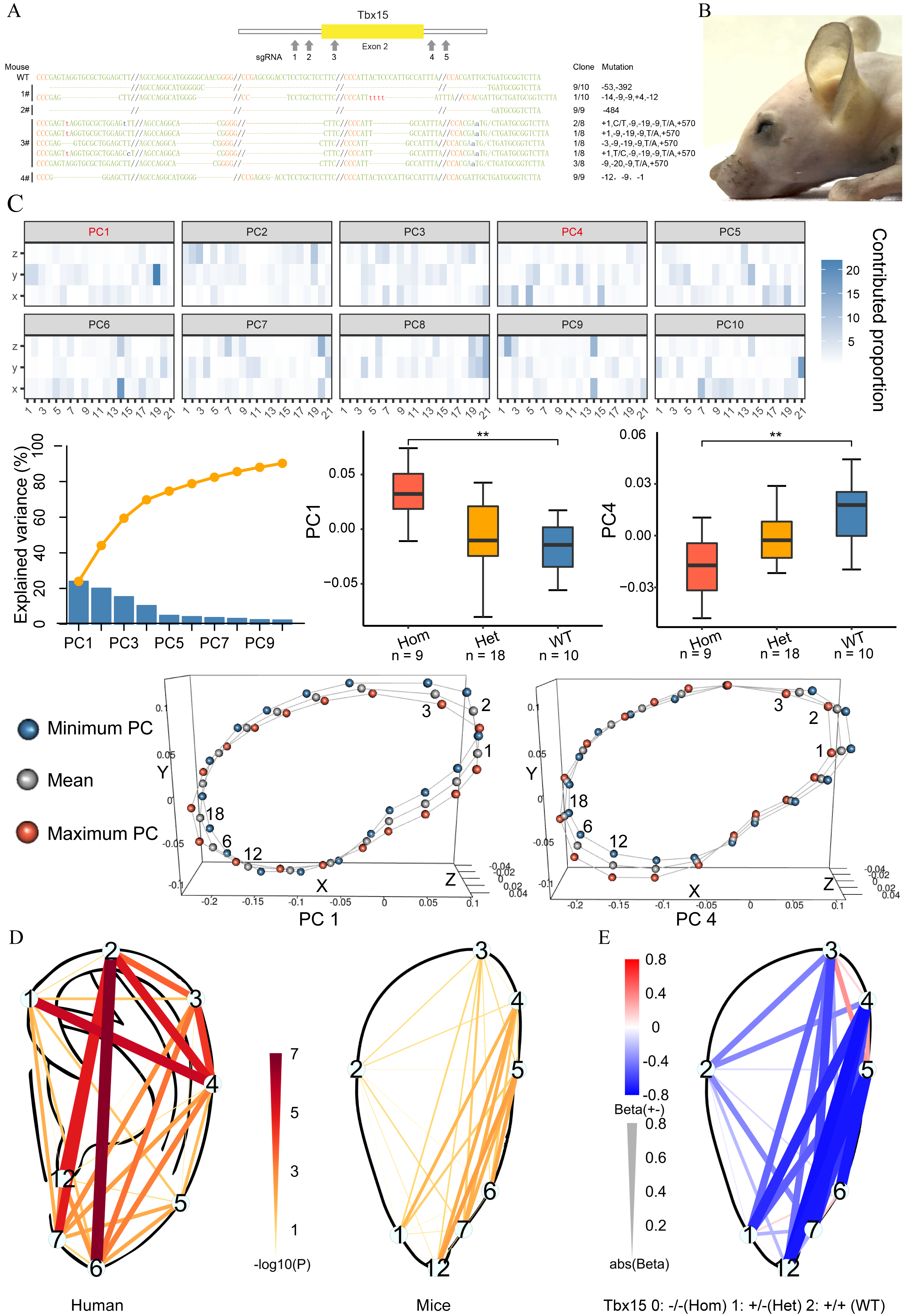

Supplement: S9 Fig — Homozygous Tbx15-/- mutant mice (N = 9, 9 weeks), heterozygous Tbx15+/- (N = 18, 9 weeks) and C57BL/6 WT+/+ control mice (N = 10, 9 weeks) were compared for ear and body morphological differences. (A) The schematic diagram of the one-step CRISPR/Cas9 technology used in Tbx15 knockout mice. (B) Example of left profile craniofacial photo of Tbx15-/- mutant mice with removal hair. (C) The principal component analysis for 21 landmarks of Tbx15-/- mutant mice, heterozygous Tbx15+/- and WT+/+ mice. The upper layer shown the detailed contribution proportion of 21 landmarks to the first 10 principal components. The middle layer shown the screenplot of first 10 PCs, the significant association between the genotype and PCs which including PC1 and PC4 (* P < 0.05, ** P < 0.01, *** P < 0.001). The bottom shown the maximum PC1-, minimum PC1-,maximum PC4-, minimum PC4-, and mean ear shapes. (D) The pattern of genetic association in humans (left) and in mice (right). (E) Effect of Tbx15 knock-out on ear phenotypes in mice (blue for effect of heterozygote mutant and red for wildtype). (TIF) [file pgen.1010786.s009.tif]

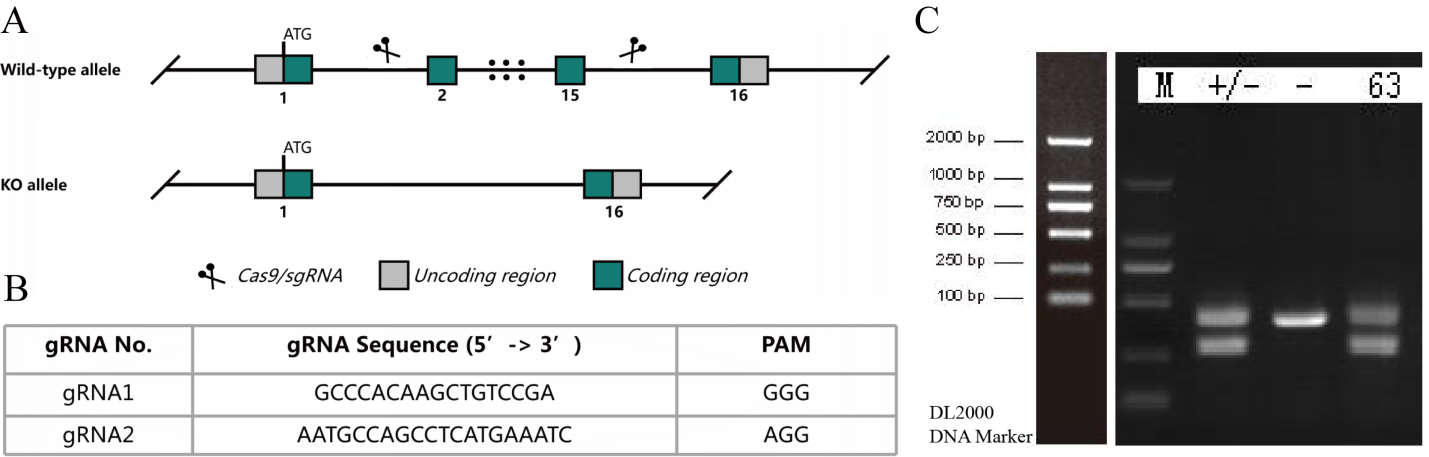

Supplement: S10 Fig — (A) The schematic diagram. (B) the sequence of gRNA. (C) Genotype was identified by PCR (Positive 251kb (loss-function), negative 402kb (wild type), heterozygous including 251kb and 402kb). (TIF) [file pgen.1010786.s010.tif]
