# Supplementary material for: Combined genome-wide association study of 136 quantitative ear morphology traits in multiple populations reveal 8 novel loci
Source: PLoS Genet. 2023 Jul 17;19(7):e1010786. doi: 10.1371/journal.pgen.1010786 (PMC10351707; doi:10.1371/journal.pgen.1010786)

Supplementary Figure S6: 16 ear-associated loci we identified in our current MinGWAS and C-GWAS.

The first 8 (A-H) were novel loci, others 8 were previously reported loci (I-P). Each figure includes three figures, LocusZoom (up) shows regional association plots for the top-associated ear phenotype (p values in CGWAS, except for the 6q21 PRDM1/ATG5, which solely identified by meta-analysis) with candidate genes aligned below according to the chromosomal positions (GRCh37.p13) followed by the linkage disequilibrium (LD) patterns (r2) of European. Ear map (left lower) shows the association (p values in Meta-analysis) between all ear phenotypes (P < 1e-3) and top-SNP identified in our analysis. Effect plot (right lower) shows effect sizes for the effect allele of top-SNPs from the association with top-associated ear phenotype in all 5 GWASs and meta-analysis.


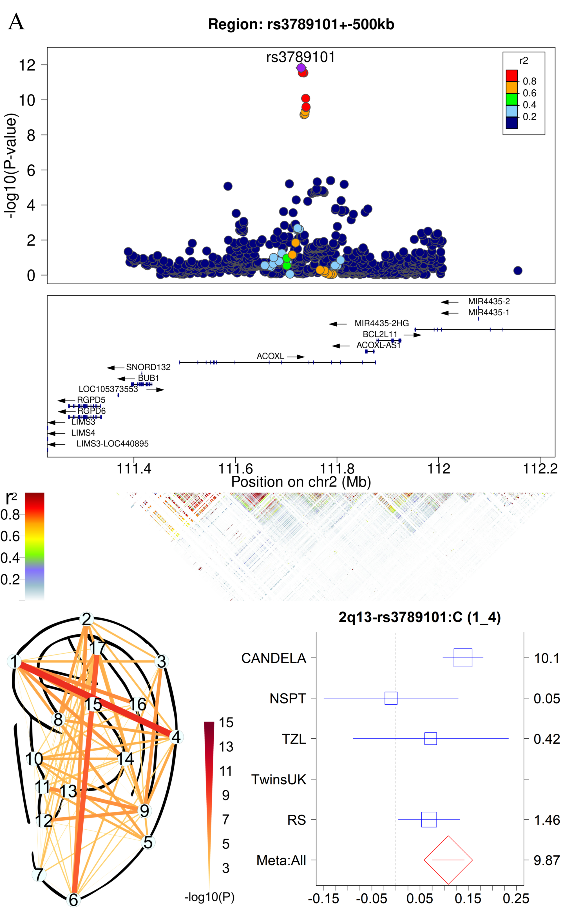

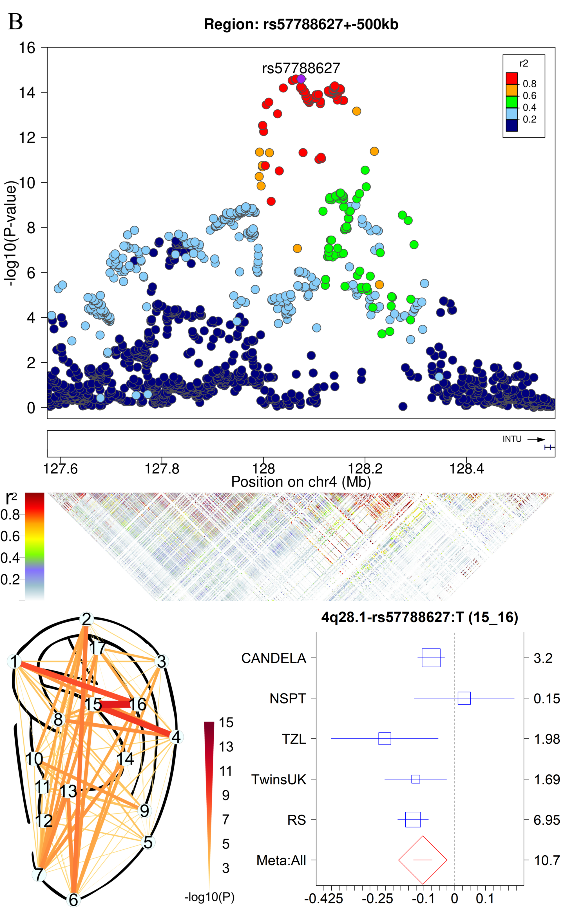


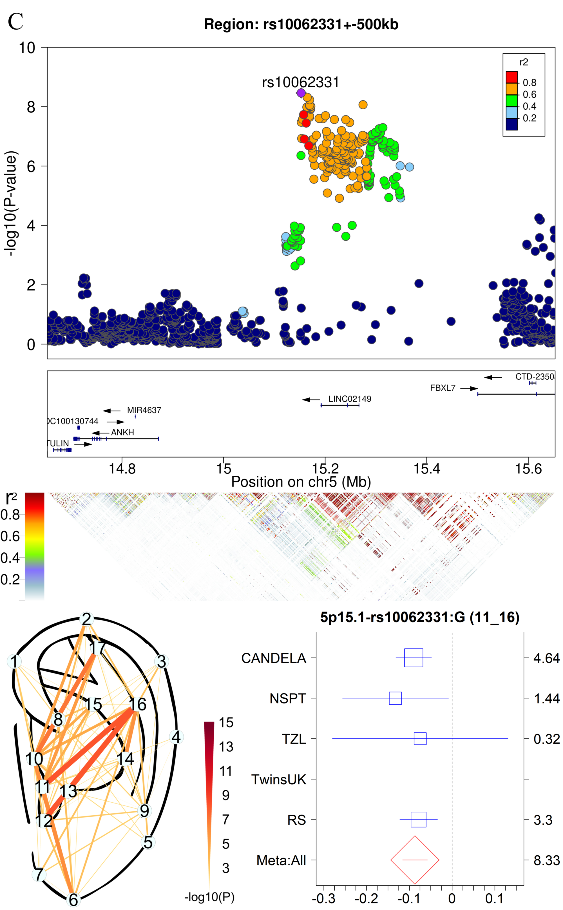

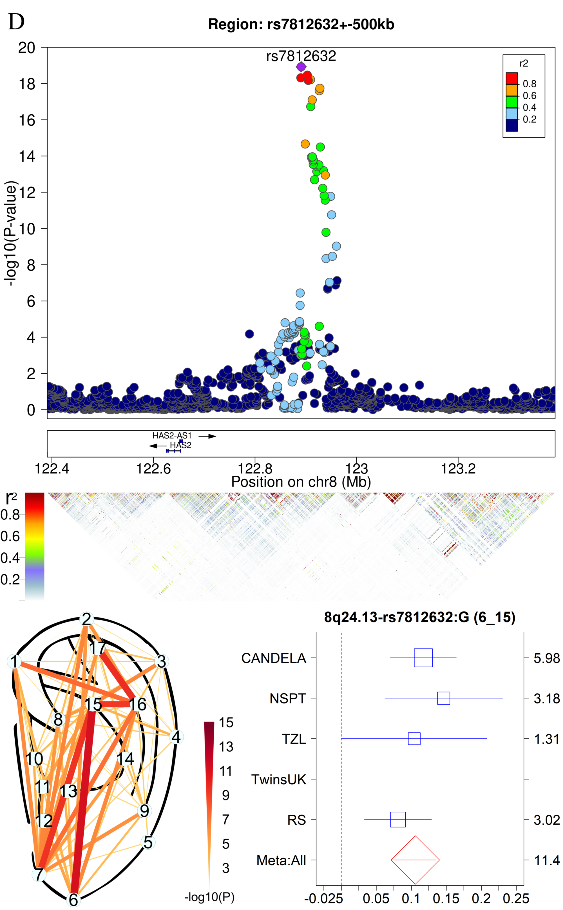


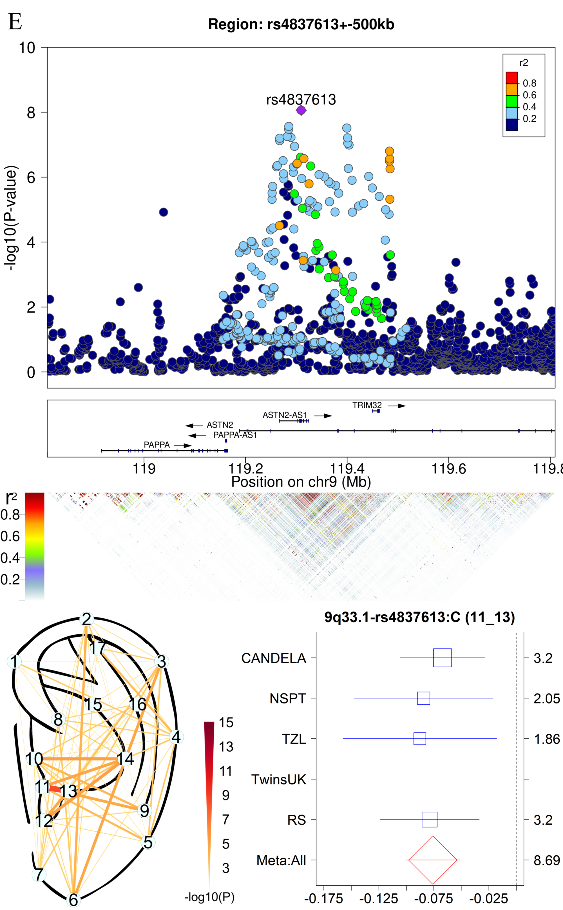

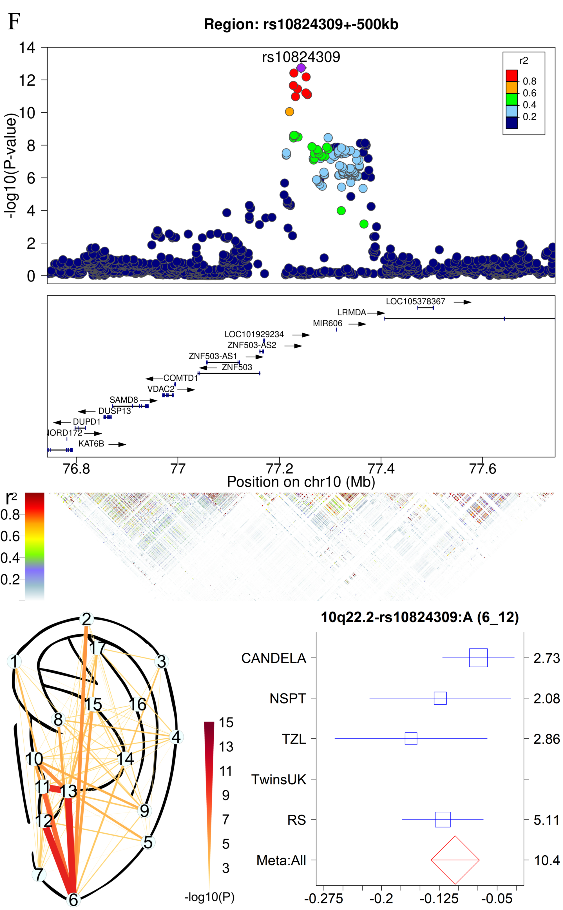


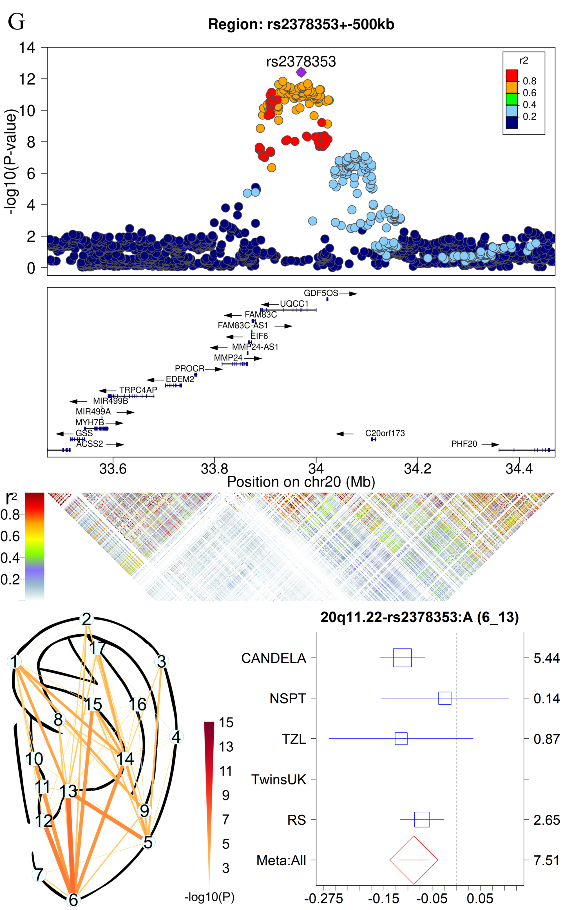

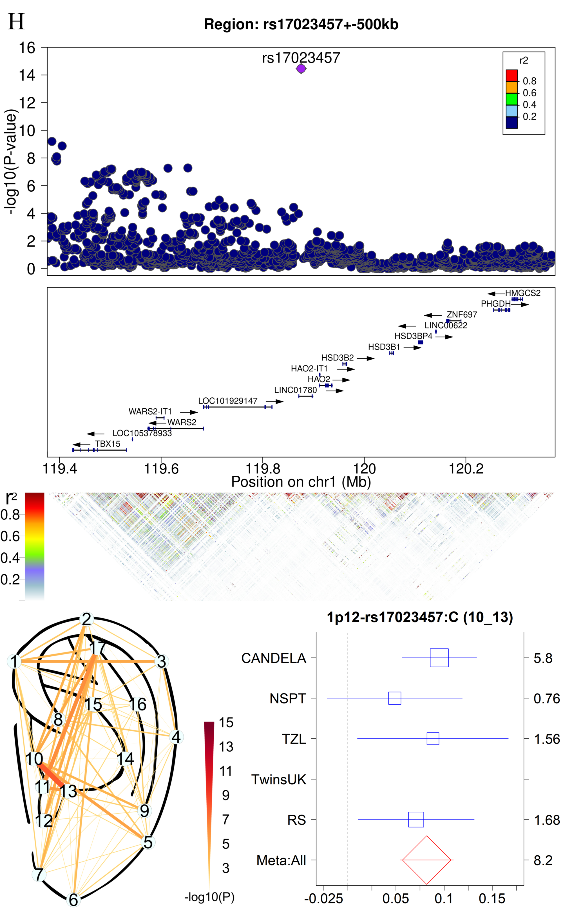


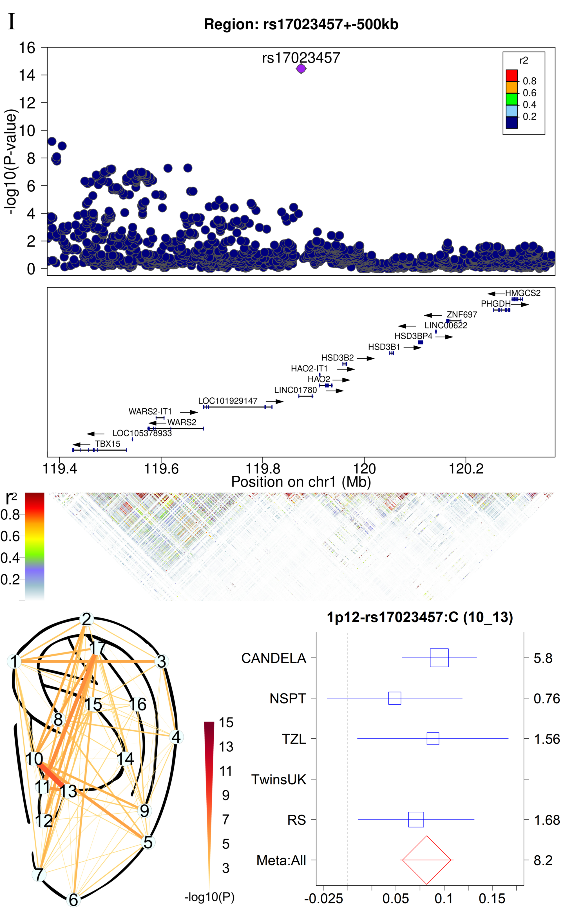

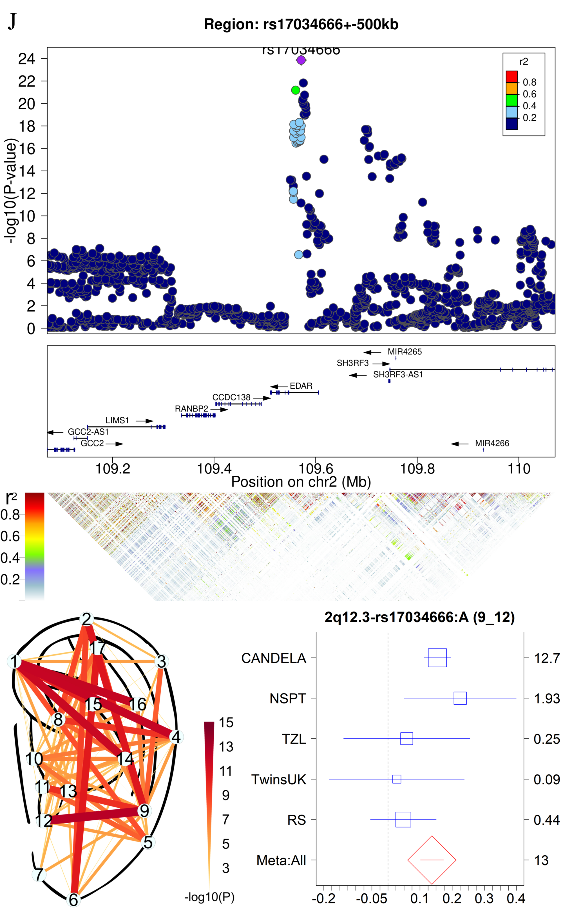


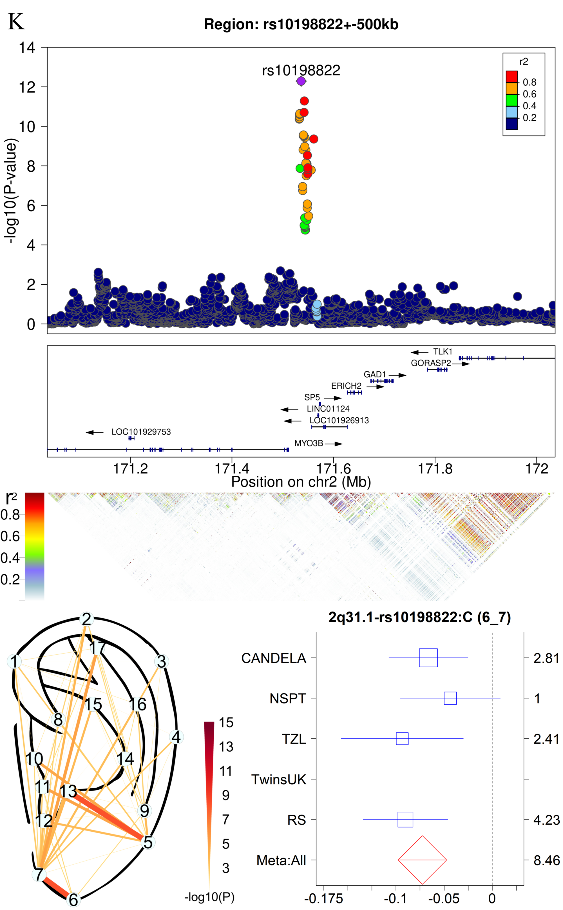

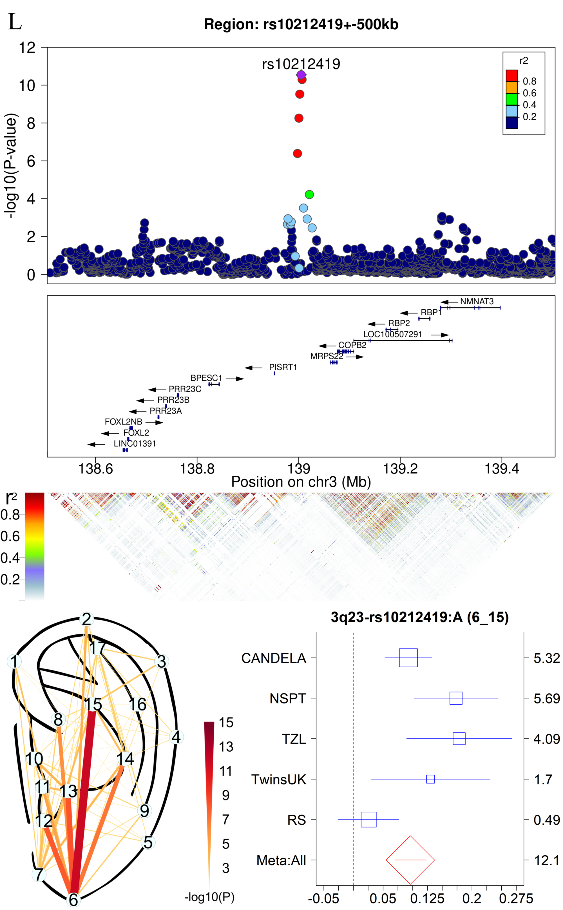


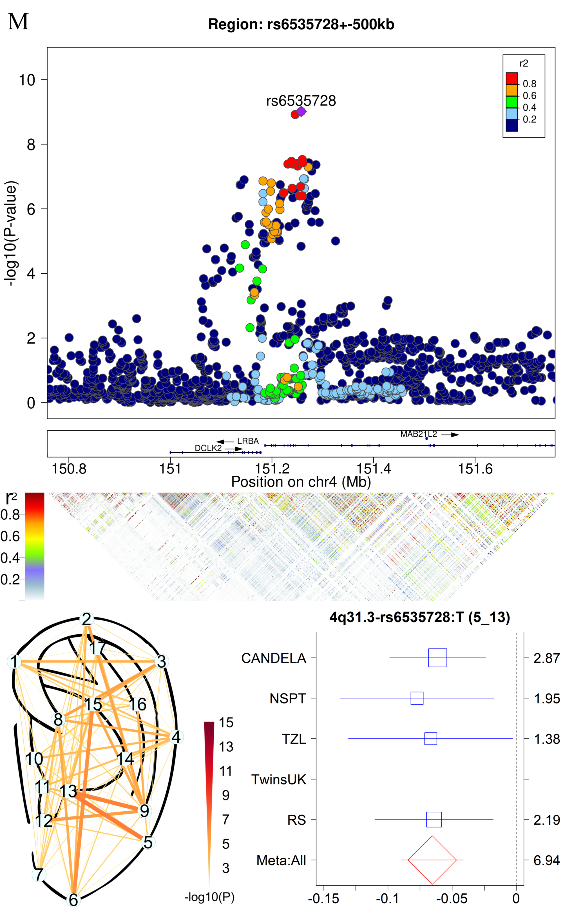

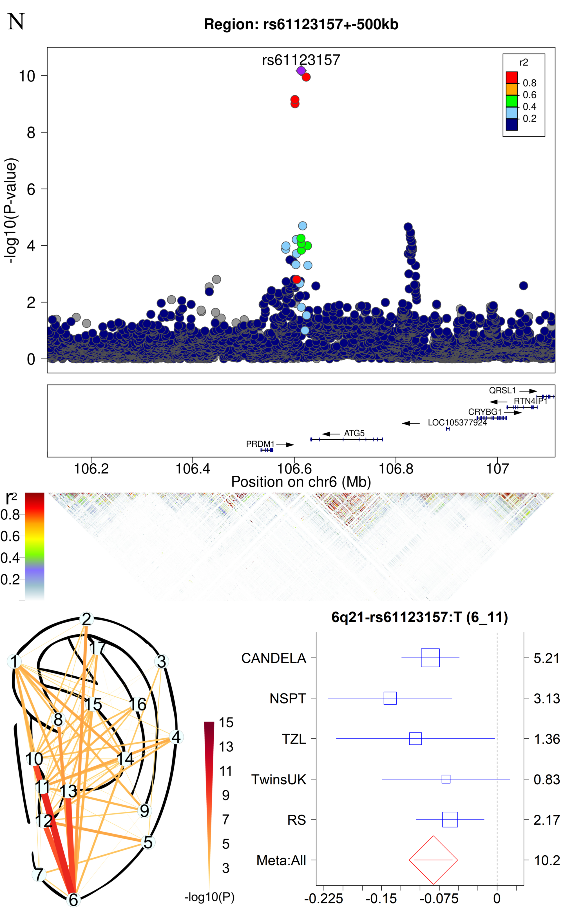


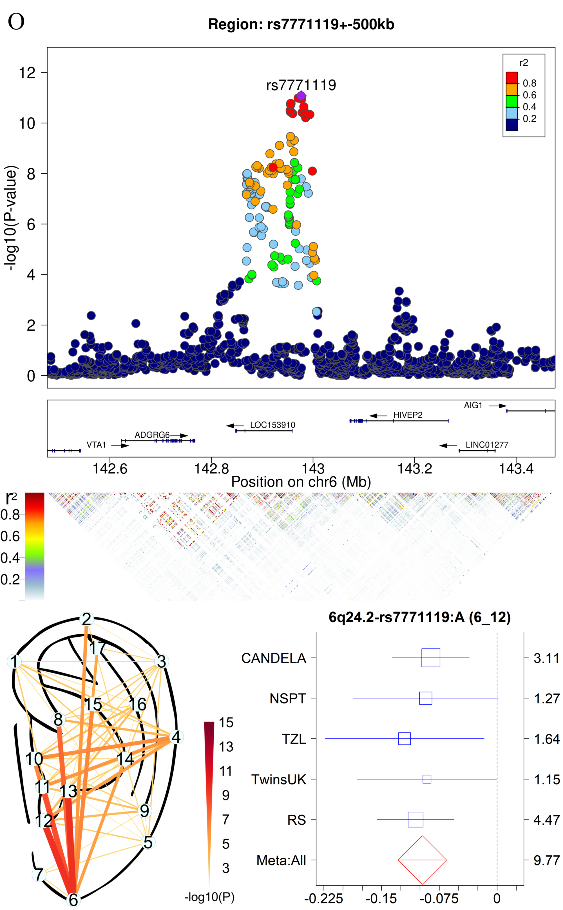

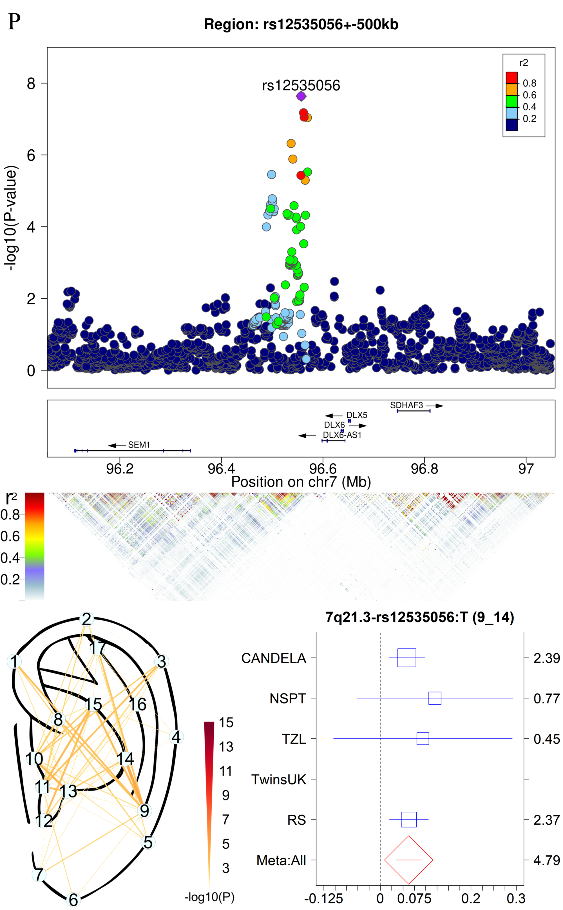

Supplement: S6 Fig — The first 8 (A-H) were novel loci, others 8 were previously reported loci (I-P). Each figure includes three figures, LocusZoom (up) shows regional association plots for the top-associated ear phenotype (p values in CGWAS, except for the 6q21 PRDM1/ATG5, which solely identified by meta-analysis) with candidate genes aligned below according to the chromosomal positions (GRCh37.p13) followed by the linkage disequilibrium (LD) patterns (r2) of European. Ear map (left lower) shows the association (p values in Meta-analysis) between all ear phenotypes (P < 1e-3) and top-SNP identified in our analysis. Effect plot (right lower) shows effect sizes for the effect allele of top-SNPs from the association with top-associated ear phenotype in all 5 GWASs and meta-analysis. (DOCX) [file pgen.1010786.s006.docx]
