# Supplementary material for: Combined genome-wide association study of 136 quantitative ear morphology traits in multiple populations reveal 8 novel loci
Source: PLoS Genet. 2023 Jul 17;19(7):e1010786. doi: 10.1371/journal.pgen.1010786 (PMC10351707; doi:10.1371/journal.pgen.1010786)
Supplement: Attachment — Submitted filename: response_to_reviewers.docx [file pgen.1010786.s025.docx]

Dear Editor,

Thank you for your interest in our work. First of all, the new method C-GWAS used in this study has been published very recently in nature communications, so we cite this paper in our revised manuscript. We appreciate the detailed and constructive comments provided by the reviewers We have carefully revised the manuscript based on the reviewers' feedback. Because the modifications regarding the logic and language were rather substantial, we only emphasized modifications that pertain to the reviewers’ comments, added analysis and findings. Below, you will find our response to the reviewers' comments, point by point.

Reviewer #1: This is an impressive paper representing an enormous amount of work by many parties. The methods and results are interesting and convincing. Ideally there would be more detail but the paper is already long. My one request is that they should include a photo or a very good drawing to show what Darwin's tubercle is. My only other request is that the paper should be given a final read by a native English speaker since at the moment there are many spelling errors and infelicities of expression which detract from the overall quality.

We appreciate the reviewer's interest in our work and the grate suggestion. We have expanded our results on Darwin's tubercle (lines 208-210) and included a photo for illustration (S7 Fig, shown in below). Additionally, we have thoroughly revised the main text with the help of native English speakers for language improvement. We believe these revisions have significantly improved the logic and language. Because the modifications regarding the logic and language were rather substantial, we have only highlighted modifications that relate to reviewer’s comments, new analysis and findings.

**S7 Fig.** Various degrees of Darwin's tubercle.

Reviewer #2: In this manuscript, Li et al use quantitative phenotyping approaches as well as a new method for combining GWAS summary statistics from multiple traits to discover known and novel loci influencing variation in ear morphology in individuals of multiple ancestries. They provide evidence that candidate genes near these loci are preferentially expressed in cranial neural crest cells and having functions related to skeletal, craniofacial, and ear development. They show that one of the candidate genes, Intu, affects ear morphology upon heterozygous loss in mice.

Overall, this is a reasonably well-conducted study, with no fundamental claims that are unsupported by data. The deep learning phenotyping pipeline and C-GWAS methodological development could be potentially useful for others in the field. The measurement of ear morphology in knockout mice is another strength. One weakness is that there is a lack of independent replication, but the authors at least note this in their discussion. This is important information for readers to know when interpreting this manuscript. Another general point which confuses me is the authors’ use of the term “ectoderm-derived” when referring to traits such as ear morphology, face shape, etc. While it is true that many (although not all) of the contribution to these structures comes from ectoderm (i.e. CNCCs and surface ectoderm), there are numerous other tissues/traits that are also ectoderm-derived (it is, after all, one of the three germ layers) that presumably do not have the same genetics. If the authors are intending to make a more specific point about, for eg, surface ectoderm being a major contributor, then that should be stated, but in my opinion that is not well-supported here. It would be simpler to refer to these related traits as “craniofacial traits” or something along those lines.

We appreciate the reviewer's interest in our work and the detailed suggestion. We replaced “ectoderm” with “surface ectoderm” throughout the manuscript expect for the more specific part replaced with “craniofacial traits” (lines 264,283-286).

There are several claims made in the manuscript which in my opinion should be adjusted or supported with additional analyses:

Line 85, it is somewhat of an exaggeration to say that this study “corrects the long-term view that ear phenotypes are genetically simple traits.” Indeed previous ear morphology GWAS had already found 8 loci, and combined with the fact that related traits such as face and cranial shape have hundreds of associated loci and are clearly genetically complex, there is very little evidence for such a view of ear morphology being simple.

Thank for your detailed suggestion. We have revised and adapted the statements in the manuscript accordingly (lines 84-86), details following:

“These genetic loci can serve as candidates for future research to understand the function of these genes. Additionally, we have demonstrated the impact of two ear-associated loci on ear morphology in gene-edited mice. This study provides new insights into the complex genetics of the human ear.”

The authors claim (lines 148-149) that their deep learning phenotyping pipeline “convincingly” outperforms human perception in ear landmarking. This is not well supported by the data. The left-right ear correlation (which the authors consider a gold standard of accuracy) for deep learning is 0.52 with sd 0.1, while the correlation for human raters is 0.44 with sd 0.2. Thus human raters are well within one sd with respect to this measure of accuracy. All that can be said from this is that the performance of the deep learning pipeline is roughly similar to that of human raters. This is not a problem (there is no need to demonstrate superior accuracy) but this claim should be rephrased accordingly.

Thank for your detailed suggestion. We have revised and adapted the statements in the manuscript accordingly (lines 145-147), details following:

“This demonstrated that the deep learning approach on ear phenotyping that we used in this study on the full set of facial images had at least an equal performance in the accuracy of ear landmarking compared to human perception, while also being more efficient.”

The expression analysis of genes near the 16 loci is not convincing. An appropriate background set would be genes near to (or closest to) a random set of control, frequency-matched SNPs that are the same size as the true ear morphology-associated SNPs. Such sets can easily be obtained using a tool such as SNPsnap. Expression within CNCCs, as well as comparing CNCCs to other tissues, should be performed with such a background set. The authors should also perform heritability enrichment analysis (i.e. stratified LD score regression) for the univariate ear morphology traits (only TwinsUK perhaps) with respect to active regulatory regions from CNCCs and other cell types, all of which are publicly available. This would be much more convincing when claiming the importance of certain cell types for ear morphology and would avoid the issue of analyzing small sets of genes/SNPs for statistical significance.

We appreciate the reviewer's interest in our work and the grate suggestion. Regarding the issue about the appropriate background set, as your advice, we used the tool “SNPsnap” for attaining the genes near the random and frequency-matched SNPs as control set.

we revised our results as (lines 304-310):

“We compared the expression of 16 genes located near the 16 ear-associated SNPs with a random set of 16 genes selected near randomly chosen and frequency-matched 16 SNPs in CNCCs and 49 other cell types for 10000 replicates. Compared with the randomly selected gene sets, this set of 16 targeted genes near the ear-associated SNPs had significantly higher expression in 23 types of cells (*P* < 0.05, **Fig 2A**), including those involved in the formation of constituent of ear, such as CNCCs, articular chondrocyte, osteoblast, skin fibroblast, and adipose mesenchymal stem cell.”

And corresponding methods part (lines 614-616):

“We attained all annotated nearest genes of the 1e-4 sets of matched SNPs for 16 lead SNPs using SNPsnap [63], the 16 genes with the median expression value separately in 50 cell types were as the correspondent control genes.”

Regarding the necessary of performing heritability enrichment analysis, we added the heritability enrichment analysis by using the method stratified LD score regression.

We add the following to our result section (lines 310-316):

“In addition, we performed a heritability enrichment analysis using the S-LDSC method [38] with respect to active regulatory regions based on our C-GWAS results of Europeans (RS and TwinsUK). This analysis showed that the S-LDSC coefficient Z-scores in CNCCs, osteoblast primary cells, adipose derived mesenchymal stem cell cultured cells, mesenchymal stem cell derived chondrocyte cultured cells, and HSMM cell derived skeletal muscle myotubes cells all ranked within the top 5% in a total of 101 types of cells (**Fig 2C**).”

We add the following to our method section (lines 616-625):

“Partitioned heritability enrichments based on 4-stages of CNCCs [64] and 97 cell types form Roadmap Epigenomics resource [65, 66]. The annotations for embryonic craniofacial tissues and other cell types refer the previous study [67] using S-LDSC [38]. For embryonic craniofacial tissues, we combined all regions with the following annotations from the 25-state chromHMM model: ‘Enh’, ‘TxReg’, ‘PromD1’, ‘PromD2’, ‘PromU’ and ‘TssA’. For other cell types, we combined all regions with the following annotations from the 15-state chromHMM model: ‘1_TssA’, ‘2_TssAFlnk’, ‘7_Enh’ and ‘6_EnhG’. Each annotation was individually added to the baseline LD model [38]. Also, we use the C-GWAS summary data for 136 meta-analysis in European populations including RS and TwinsUK.”

Some of the analyses of ear morphology, in particular comparing PC loadings between genotypes, is not entirely statistically sound. Comparing PC loadings between samples upon which PC was initially performed will exaggerate statistical significance (and indeed even with this PC2 is only weakly associated with genotype). I believe that there are significant (albeit weak) differences in ear morphology between Intu hets and WT, as indicated by the univariate landmark analysis in 3F. If the authors wish to perform an integrated morphological analysis by genotype, they should use a generalized Procrustes analysis framework (such as the one available in the R package geomorph), which would allow them to test for overall shape changes as a function of genotype. The effects of Tbx15 KO are stronger, but the manuscript would benefit from similar analyses for Tbx15 het/KO mice as well.

We appreciate the reviewer's interest in our work and the grate suggestion. We have carefully updated the analysis of ear morphology. More details please see in below.

We add the following to our result section (lines 332-341; 348-351):

“Quantitative assessment of mouse ear shape (assessed by PC analysis of 21 three-dimensional landmark coordinates) revealed significant differences (linear regression *P*_PC3_ = 1.5e-3) (**Fig 3C**) between the heterozygous *Intu* littermates and WT mice. The *Intu* genotype significantly associated with D6_14 (*P* = 2.1e-3, Beta = 1.78) and D3_6 (*P* = 3.3e-3, Beta = 1.74) (**Fig 3E and S11 Table**) after FDR correction. Notably, in humans, the top *INTU* variant rs57788627 was nominally significantly associated with ear phenotypes L3-L6 and L3-L7, which respectively corresponds to D6_14 and D3_6 in mice (*P* = 1.9e-5, Beta = 0.07; *P* = 8.7e-5, Beta = 0.06) (**Fig 3E and S11 Table**). Overall, the ears of the heterozygous *Intu* mutant mice were shorter than those of the WT mice consistently show in the result of PC3 and distance phenotypes (**Fig 3D-F**).”

“In addition to the distinct “droopy ear” feature, the *Tbx15* genotype was significantly associated with the ear landmarks PC1 (*P* = 3.2e-3) and PC4 (*P* = 1.9e-3) (**S9 Fig C**), and the variation for two PCs shown the shift of landmarks. Overall, the ears of the *Tbx15* mutant mice were obviously longer and wider than those of the *WT* mice (**S9 Fig E-F**).”

We add the following to our method section (lines 648-657):

“The R package ‘geomorph’ were used for series of analysis (https://github.com/geomorphR/geomorph). GPA was used to remove the effects of translation, rotation and scaling. After superimposition of the GPA-adjusted coordinates, only the shape component remained in the aligned specimens. Mice with the outlier in PC1 were excluded then the GPA and PCA were redone. The first 10 PCs were used to represent dominant but different dimensional variations of all landmarks. The ear shape visualization of shape variation based on the maximum and minimum PC compare to mean ear shape. The inter-landmark also distances also were visualized (**S13 Table**). Z-transformed variables were used for association analysis. The effect of per mutant allele on ear shape was tested using linear regression with covariate sex, weight and body length. Multiple testing was corrected using FDR method.”
