# Supplementary material for: Combined genome-wide association study of 136 quantitative ear morphology traits in multiple populations reveal 8 novel loci
Source: PLoS Genet. 2023 Jul 17;19(7):e1010786. doi: 10.1371/journal.pgen.1010786 (PMC10351707; doi:10.1371/journal.pgen.1010786)
Supplement: Attachment — Submitted filename: response_to_reviewers.docx [file pgen.1010786.s026.docx]

Dear Editor,

Thank you for considering our revised manuscript. We appreciate the constructive comments provided by the reviewer. We have carefully revised the manuscript based on the reviewer’s feedback. The modifications that pertain to the reviewer’s comment were highlighted. We believe these changes have significantly improved the accuracy and clarity of our findings. We look forward to hearing from you. Below, you will find our response to the reviewer’s comment.

Reviewer #2: The authors have addressed all of my comments, except for one important error in which they refer to embryonic craniofacial tissue samples (CS13, 14, 15, 17 in the S-LDSC analysis in Fig 2c) as CNCCs. These are not CNCCs, they are primary tissue from embryonic craniofacial regions which will be a mix of many cell types. More details are found in the cited paper (Wilderman et al 2018). For CNCCs the authors should analyze ATAC-seq/H3K27ac peaks from hESC-derived CNCCs, available from Prescott et al (ref 61 in the paper).

We appreciate the reviewer for pointing this out. Following their advice, we downloaded the ATAC-seq/H3K27ac dataset of CNCCs (Prescott et al 2015) and called peaks and inferred enhancers including strong enhancers. All enhancers have been annotated and subjected to S-LDSC analysis accordingly. The specific modifications in the text are shown below.

We revised our results as (lines 252-256):

“This analysis showed that the S-LDSC coefficient Z-scores in mesenchymal stem cells, osteoblast primary cells, adult dermal fibroblast primary cells, adipose derived mesenchymal stem cell cultured cells, mesenchymal stem cell derived chondrocyte cultured cells, all ranked within the top 5% in a total of 102 types of cells and CNCCs slightly behind them (ranked 9th) (Fig 2C).”

And corresponding methods part (lines 557-565, please note that we also add a supplementary table about the list of enhancer of CNCCs):

“Partitioned heritability enrichments based on CNCCs [61], 4-stages of embryonic craniofacial tissues [64] and 97 cell types form Roadmap Epigenomics resource [65, 66]. The annotations for CNCCs, embryonic craniofacial tissues and other cell types refer the previous study [67] using S-LDSC [38]. For CNCCs, we downloaded and processed H3K27ac and ATAC data, and all data from different replicates. Peaks were called using MACS2 [68]. We iteratively obtained and combined the most reliable peaks based on a 50% peak overlap rate for all replicates from of H3K27ac and ATAC data using BEDtools. [69]. Based on the combined peaks, we used ROSE [70] to infer enhancers including super-enhancers and annotated all enhancer regions (S14 Table).”

Corresponding Figures (Fig 2):
